# Preoperative radiological characterization of hepatic angiomyolipoma using magnetic resonance imaging and contrast-enhanced ultrasonography: a case report

**DOI:** 10.1186/1752-1947-5-481

**Published:** 2011-09-26

**Authors:** Stephanie Krebs, Irene Esposito, Christian Lersch, Jochen Gaa, Roland M Schmid, Oliver Ebert

**Affiliations:** 1 Medical Clinic, Klinikum rechts der Isar, Technische Universität München, Munich, Germany; 2 Department of Pathology, Klinikum rechts der Isar, Technische Universität München, Munich, Germany; 3 Department of Radiology, Klinikum rechts der Isar, Technische Universität München, Munich, Germany

## Abstract

**Introduction:**

A hepatic angiomyolipoma is a rare benign tumor of the liver composed of a mixture of smooth muscle cells, blood vessels and a variable amount of adipose tissue. Differentiating them from malignant liver tumors can often be very difficult.

**Case presentation:**

We report the case of a 43-year-old Caucasian man presenting with a large liver mass in the right lobe. The results of magnetic resonance imaging and contrast-enhanced ultrasonography were consistent with a well-demarcated adipose tissue- containing tumor, showing prolonged hyperperfusion in comparison with the surrounding liver tissue. Surgery was performed and the diagnosis of hepatic angiomyolipoma was made with histopathology.

**Conclusion:**

Preoperative radiological characterization using magnetic resonance imaging and contrast-enhanced ultrasonography may improve diagnostic accuracy of hepatic angiomyolipoma. Identification of smooth muscle cells, blood vessels and adipose tissue with a positive immunohistochemical reaction for HMB-45 is the final evidence for an angiomyolipoma.

## Introduction

An angiomyolipoma is a rare benign mesenchymal tumor composed of a mixture of smooth muscle cells, blood vessels and a variable amount of fat. The appearance of an angiomyolipoma has often been reported in the kidney but rarely in the liver. The first hepatic angiomyolipoma was described by Ishak in 1976 [1]. It can be found in both men and women, most commonly in adult women. The majority of patients with hepatic angiomyolipoma are asymptomatic. Others suffer from abdominal pain and mild fever [2]. There is no histological difference between an angiomyolipoma of the liver or the kidney. A differentiation of malignant liver tumors is often very difficult because of the high variability of the fat proportion.

### Case presentation

**Figure 1:**
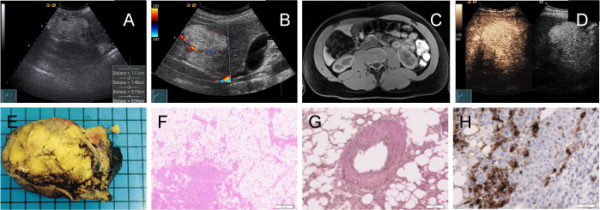
**Radiological and histopathological characterization of hepatic angiomyolipoma**. (A) Detection of a hepatic lesion by abdominal ultrasonography. (B) Color Doppler examination showing peripheral hypervascularization. (C) Typical MRI aspects of hepatic angiomyolipoma. (D) CEUS findings. (E) Gross pathological findings after surgery. (F) Histological demonstration of mostly adipose tissue. (G) Demonstration of epithelioid cells typically arranged around blood vessels. (H) Positive immunohistochemistry for the melanoma marker HMB-45.

A 43-year-old Caucasian man was found to have a large echogenic mass in his right liver lobe by abdominal ultrasonography. The hepatic lesion measured about 5 × 9 cm with a well-demarcated border (Figure 1A). Color Doppler imaging showed intense vascularization in the periphery of the tumor (Figure 1B). Five months prior to admission, our patient had noticed chronic fatigue, a loss of efficiency and abdominal stress under his right costal arch. A physical examination was unremarkable. Serum biochemistry tests were without any pathological findings. The serologic assay for viral hepatitis B and C were negative. The serum alpha-fetoprotein level was 1.5 ng/mL (normal range < 6 ng/mL). Magnetic resonance imaging (MRI; 1.5T Magnetom Avanto, Siemens Medical Solutions, Erlangen, Germany) of the liver mass using a native fat-suppressed T1-weighted gradient echo sequence showed a fat-equivalent signal behavior comparable to that of the subcutaneous adipose tissue (Figure 1C). A contrast-enhanced ultrasonography (CEUS; Acuson Sequoia, Siemens Medical Solutions) was performed for further information. It revealed a well-demarcated hyperechoic liver mass in segment VI, measuring 9.7 × 9.2 × 5.4 cm in maximum diameter with a hypoechogenic portion. The margin was well defined and smooth. The contrast agent (SonoVue, Bracco International, Amsterdam, Netherlands) showed a hyperperfusion from the early to the late vascular phase in comparison with the surrounding liver tissue. There was no sign of "halo" or "nodule-in-nodule" formation (Figure 1D).

After presenting all imaging results in an interdisciplinary liver conference, an atypical resection of the right lobe of his liver was recommended. Our patient underwent surgery without any problems. After nine days, he left the hospital in good clinical condition. The macroscopic examination revealed a solitary tumor with a mottled yellow color and a soft consistency in the right lobe of his liver (Figure 1E). The microscopic examination showed that the tumor was well circumscribed but non-encapsulated and consisted mostly of adipose tissue with solid areas of elongated and rounded epithelioid cells, often arranged around thick-walled blood vessels (Figure 1F and 1G). In the solid areas, the cells expressed smooth muscle actin and the melanoma marker HMB-45 (Figure 1H). Extramedullary hematopoiesis was not observed. A diagnosis of hepatic angiomyolipoma was made. Six months after surgery our patient is in good clinical condition. Chronic fatigue and loss of efficiency have disappeared. Abdominal ultrasonography and serum biochemistry tests were without any pathological findings.

## Discussion

Hepatic angiomyolipoma has been considered as a benign disease in the past. However, spontaneous tumor rupture, malignant transformation and recurrence after primary resection, as well as concurrent hepatic angiomyolipoma and hepatocellular carcinoma, have been reported [3,4]. Surgical resection should be considered for all symptomatic patients. Conservative management with close follow-up is suggested in patients with asymptomatic tumors, good compliance and absence of hepatitis virus infection, as well as small hepatic angiomyolipoma (< 5 cm) that are diagnosed through fine-needle aspiration biopsy [5].

Because of the variable amount of adipose tissue in hepatic angiomyolipoma, it is sometimes difficult to distinguish between benign and malignant tumors. It is possible to misdiagnose hepatic angiomyolipoma as lipoma, hepatocellular adenoma or carcinoma, sarcoma or metastasis before surgery. Contrast-enhanced spiral computed tomography shows a marked enhancement in the arterial and the portal venous phase of the soft tissue components [5]. Recently, CEUS has been shown to demonstrate typical imaging characteristics of hepatic angiomyolipoma; an inhomogeneous hyperenhancing pattern in arterial phase, prolonged hyperenhancing during portal and late phase, with a smooth and well-defined margin [6]. MRI is considered the best imaging method to identify an angiomyolipoma. It shows a hyperintensity on T2-weighted images and a hypointensity on T1-weighted images, according to the amount of adipose tissue [5].

## Conclusion

Although the combination of various imaging modalities increases preoperative diagnostic accuracy it is reported to be less than 25% [7]. A definitive diagnosis is only given by a histological examination after surgery. Identification of smooth muscle cells, blood vessels and adipose tissue with a positive immunohistochemical reaction for HMB-45 is the final evidence for an angiomyolipoma [8].

## Consent

Written informed consent was obtained from the patient for publication of this case report and any accompanying images. A copy of the written consent is available for review by the Editor-in-Chief of this journal.

## Competing interests

The authors declare that they have no competing interests.

## Authors' contributions

SK and OE wrote the manuscript. CL was the physician who performed CEUS. JG was the radiologist in charge of the MRI. RMS gave final approval. All authors read and approved the final manuscript.

## References

[B1] Ishak KG Okuda K, Peters RL Mesenchymal tumors of the liver Hepatocellular carcinoma 1976 New York: Wiley Medical 247 307

[B2] Petrolla A Xin W Hepatic angiomylipoma Arch Pathol Lab Med 2008 132 1679 1682 18834230 10.5858/2008-132-1679-HA

[B3] Ren N Qin LX Tang ZY Wu ZQ Fan J Diagnosis and treatment of hepatic angiomyolipoma in 26 cases World J Gastroenterol 2003 9 1856 1858 12918138 10.3748/wjg.v9.i8.1856 PMC4611561

[B4] Ding GH Liu Y Wu MC Yang GS Yang JM Cong WM Diagnosis and treatment of hepatic angiomyolipoma J Surg Oncol 2011 103 807 812 10.1002/jso.21814 21283992

[B5] Yang CY Ho MC Jeng YM Hu RH Wu YM Lee PH Management of hepatic angiomyolipoma J Gastrointest Surg 2007 11 452 457 10.1007/s11605-006-0037-3 17436129 PMC1852378

[B6] Li R Zhang X Hua X Cai P Zhong H Guo Y Ding S Yan X Real-time contrast-enhanced ultrasonography of resected and immunohistochemically proven hepatic angiomyolipomas Abdominal Imaging 2010 35 676 682 10.1007/s00261-009-9592-x 20020286

[B7] Chang ZG Zhang JM Ying JQ Ge YP Characteristics and treatment strategy of hepatic angiomyolipoma: a series of 94 patients collected from four institutions J Gastrointestin Liver Dis 2011 20 65 69 21451800 10.1007/s11749-010-0230-2

[B8] Flemming P Lehmann U Becker T Klempnauer J Kreipe H Common and epithelioid variant of hepatic angiomyolipoma exhibit clonal growth and share a distinctive immunophenotype Hepatology 2000 32 213 217 10915726 10.1053/jhep.2000.9142

